# Polymorphism at the apical membrane antigen 1 locus reflects the world population history of *Plasmodium vivax*

**DOI:** 10.1186/1471-2148-8-123

**Published:** 2008-04-29

**Authors:** Priscila Grynberg, Cor Jesus F Fontes, Austin L Hughes, Érika M Braga

**Affiliations:** 1 Departamento de Parasitologia, Instituto de Ciências Biológicas, Universidade Federal de Minas Gerais, Av. Antônio Carlos 6627, 31270-901 Belo Horizonte, (MG), Brazil; 2 Departamento de Clínica Médica, Universidade Federal de Mato Grosso, Avenida Fernando Corrêa, s/n°, 78060-900 Cuiabá (MT), Brazil; 3 Department of Biological Sciences, University of South Carolina, Coker Life Sciences Bldg., 700 Sumter St., Columbia, SC 29208, USA

## Abstract

**Background:**

In malaria parasites (genus *Plasmodium*), *ama-1 *is a highly polymorphic locus encoding the Apical Membrane Protein-1, and there is evidence that the polymorphism at this locus is selectively maintained. We tested the hypothesis that polymorphism at the *ama-1 *locus reflects population history in *Plasmodium vivax*, which is believed to have originated in Southeast Asia and is widely geographically distributed. In particular, we tested for a signature of the introduction of *P. vivax *into the New World at the time of the European conquest and African slave trade and subsequent population expansion.

**Results:**

One hundred and five ama*-1 *sequences were generated and analyzed from samples from six different Brazilian states and compared with database sequences from the Old World. Old World populations of *P. vivax *showed substantial evidence of population substructure, with high sequence divergence among localities at both synonymous and nonsynonymous sites, while Brazilian isolates showed reduced diversity and little population substructure.

**Conclusion:**

These results show that genetic diversity in *P. vivax *AMA-1 reflects population history, with population substructure characterizing long-established Old World populations, whereas Brazilian populations show evidence of loss of diversity and recent population expansion.

**Note:**

Nucleotide sequence data reported is this paper are available in the GenBank™ database under the accession numbers EF031154 – EF031216 and EF057446 – EF057487

## Background

Studies of the population diversity of the malaria parasites have practical significance for the development for strategies of disease control, including vaccine development [1]. Moreover, the characterization of genes responsible for resistance to therapeutic agents by both *Plasmodium falciparum *and *Plasmodium vivax *depends on a thorough knowledge of each parasite's genetic diversity in natural populations [2]. The primary factors affecting genetic diversity at such loci are natural selection [3,4], and genetic drift. Genetic drift reflects the population history, including population bottlenecks; and it has a substantial effect on genetic diversity even at loci subject to balancing selection [5-7]. Thus, the knowledge of the parasite's population history and its genetic diversity is important for a full understanding of the epidemiology of malaria and potential response of the parasite to therapeutic strategies.

*P. vivax *is widely geographically distributed, being present in both tropical and temperate areas; this species is responsible for about 80 million annual cases of human malaria, especially in Latin America, Asia and Oceania [8]. It is the prevalent species in a great number of countries and territories, including Brazil, which accounted for about 81% of the approximately 460,000 cases reported in 2007 [9]. *P. vivax *infections rarely culminate in death of the patient but are a very important cause of morbidity and social economic loss [10]. *P. vivax *is believed to have first entered hominid populations in Southeast Asia and to have spread from there throughout the Old World based on its close relation to malaria parasites of non-human primates from Southeast Asia [11]. However, there is archaeological evidence supporting the hypothesis that both *P. vivax *and *P. falciparum *were absent from the New World in pre-Columbian times and were introduced after European colonization, presumably as a result of the African slave trade [12]. Thus *P. vivax *in the New World might be expected to have a somewhat reduced effective population size and thus reduced genetic diversity in comparison to Old World populations, as a result of founder effects in the sampling of Old World populations. Microsatellite markers have shown evidence of a substantial reduction of genetic diversity in the case of South American *P. falciparum *[13]. On the other hand, in *P. vivax*, microsatellite markers showed revealed only a rather modest reduction in genetic diversity in South America in comparison to Asia [14].

In addition to microsatellites [14-18], several polymorphic protein-coding loci have been used to examine genetic diversity of *P. vivax *populations, including genes encoding the merozoite surface proteins (MSP) [19-28]; the circumsporozoite protein (CSP) [21,24,26,28-30]; and the locus encoding apical membrane antigen 1 (AMA-1) [20,31-36]. AMA-1 is an immunogenic type 1 integral membrane protein [37-40] that is present in all *Plasmodium *species so far examined, with at least 16 cysteine residues incorporated into eight intramolecular disulfide bonds, forming the three domains of the protein [41]. This protein is synthesized late in the development of schizonts [42] during the last four hours of the erythrocytic phase [38]. AMA-1 may have a role in the beginning of the invasion process of the erythrocyte and may be directly responsible for reorientation of the merozoite; and it may initiate the junctional contact between these two cells, which is presumably dependent on Duffy binding proteins [43].

At the *ama-1 *locus in *P. falciparum*, polymorphisms occur non-randomly along the coding region, and the highest polymorphism is found in the three ectodomains, especially in domain I [44]. Moreover, the number of nonsynonymous nucleotide substitutions per nonsynonymous site (*d*_*N*_) exceeds that of synonymous nucleotide substitutions per synonymous site (*d*_*S*_), providing evidence that positive Darwinian selection has acted at this locus [4]. Combined with the evidence of a high level of polymorphism at this locus, this result supports the hypothesis that balancing selection has acted to maintain polymorphisms at this locus [4]. It has been proposed that host immune system pressure is responsible for this selection [4]; and, consistent with this hypothesis, there is evidence that polymorphisms at this locus are responsible for evasion of host antibody-mediated inhibition in *P. falciparum *[45]. In *P. vivax*, *d*_*N *_has been found to exceed *d*_*S *_in partial *ama-1 *sequences [20], suggesting that this locus is subject to balancing selection in *P. vivax *as well.

Extensive data on *ama-1 *polymorphism in *P. vivax *(*pvama-1*) have been obtained from Asia, Oceania and Africa [20,31-33,35,36], but there is a relative lack of data from South America, including Brazil. The only sequence data from Brazil involves domain I of 20 isolates; 13 polymorphic sites and eight haplotypes were reported in three Brazilian states [34].

The intention of the present study was to characterize the worldwide genetic diversity of the polymorphic domain of *pvama-1*. In addition to published sequences from throughout the world, we obtained sequences from patients in different endemic areas in the Brazilian Amazon. By examining polymorphism at this locus in Brazil and comparing it to other populations throughout the world, we tested the hypothesis that the pattern of genetic diversity at *pvama-1 *reflects population history, in particular a reduction of the effective population size of *P. vivax *in the New World. Theoretically, it is expected that effective population size will be the major factor determining gene diversity even at a locus under balancing selection, if the mutation rate and selection coefficient are constant [5-7]. A more complete understanding of the parasite's history in the New World in turn has implications for the epidemiology and control of this parasite in Brazil, where it has become a major public health problem in recent years due to the rapid peopling of the Brazilian Amazon [46-49].

## Results

We obtained 105 Brazilian *pvama-1 *sequences, covering bases 274–759 of the PH-84 isolate, corresponding to amino acids 92–253 (GenBank accession nos. EF031154 – EF031216 and EF057446 – EF057487 – see Additional file 1). The Brazilian isolates included 28 polymorphic nucleotide sites, leading to 26 amino acids replacements. Eight polymorphic sites were not previously described, including a synonymous substitution and seven non-synonymous substitutions (see Additional file 2). From 93 unique sequences, 27 haplotypes were identified (see Additional file 3). The nucleotide and haplotype diversities among Brazilian samples were 0.016620 ± 0.00073 and 0.91800 ± 0.00019, respectively.

**Figure 1:**
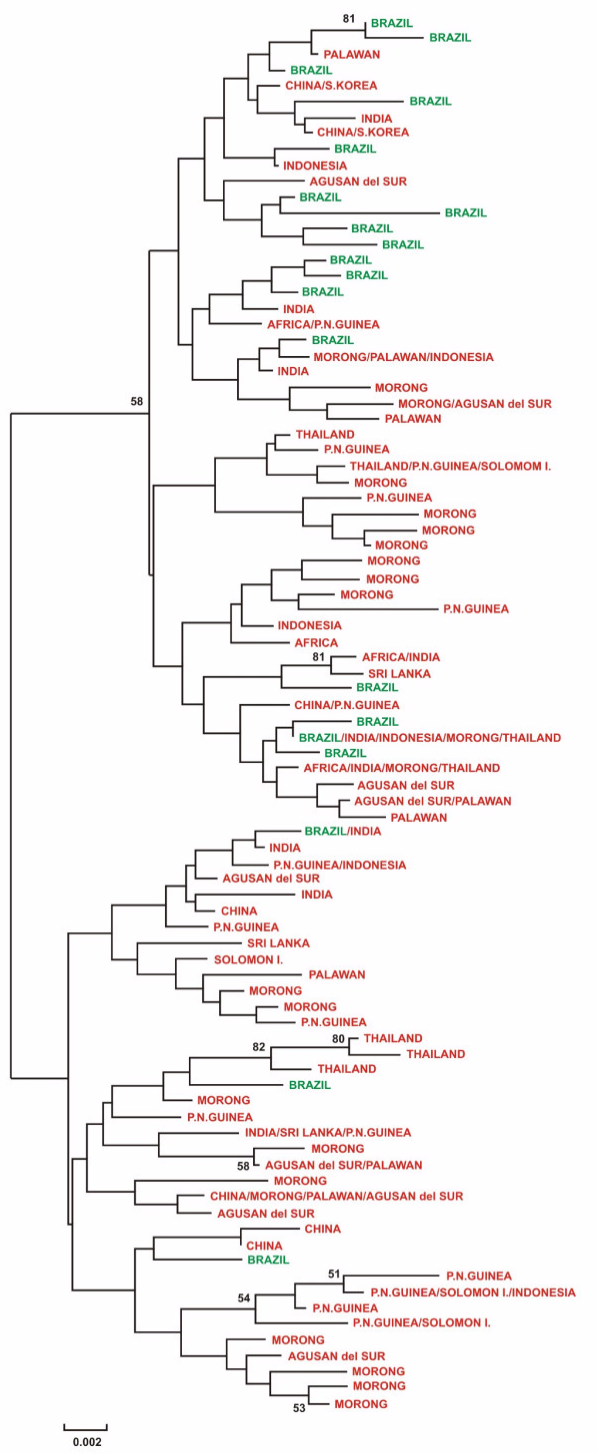
**Phylogenetic tree of unique Brazilian and worldwide *pvama-1 *sequences. **Green = South America; Red = Old Word. Numbers on the branches are percentages of bootstrap samples supporting the branch; only values ≥ 50% are shown.

A phylogenetic tree of Brazilian and worldwide sequences (Figure 1) showed no tendency toward geographic clustering of isolates. Rather, isolates from different parts of the world were found throughout the phylogenetic tree (Figure 1). The Brazilian sequences thus appeared to represent a sample from worldwide genetic diversity, rather than from any particular lineage of worldwide *pvama-1 *sequences.

**Figure 2:**
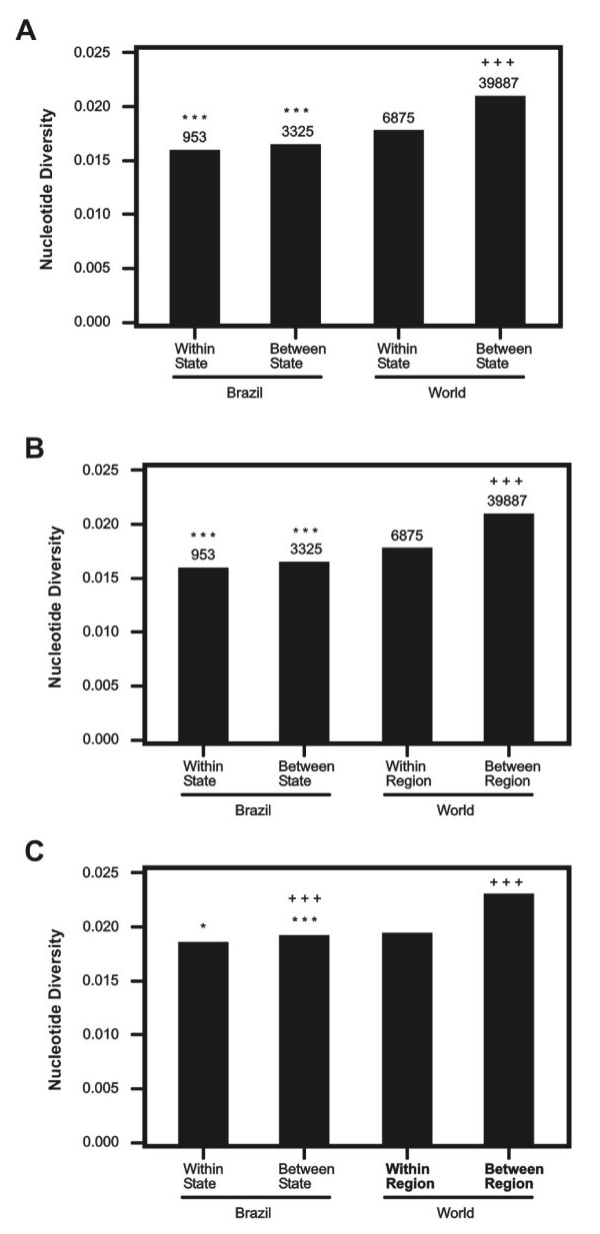
**Means of (A) π, (B) π_*S*_, and (C) π_*N *_within Brazilian states and within worldwide regions; and of (A) *d*, (B) *d*_*S*_, and (C) *d*_*N *_between Brazilian states and between worldwide regions.** Test of the hypothesis that a value for Brazil equals the corresponding value for worldwide comparisons: * P < 0.05; *** P < 0.001. Tests of the hypothesis that mean value within regions equals the corresponding value between regions: +++ < 0.001.

In order to compare nucleotide diversity within geographic regions, we computed π, π_*S*_, and π_*N *_for all pairwise comparisons within Brazilian states and within and worldwide regions (Figure 2). Likewise, we computed mean *d*, *d*_*S*_, and *d*_*N *_for all pairwise comparisons between Brazilian states and between worldwide regions. In worldwide comparisons, mean π π_*S*_, and π_*N *_within regions were always significantly lower than the corresponding values of *d*, *d*_*S*_, and *d*_*N *_between region (Figure 2). By contrast, mean π, π_*S*_, and π_*N *_within Brazilian states were not significantly different from the corresponding values of *d*, *d*_*S*_, and *d*_*N *_between states (Figure 2). Thus, these results show that *pvama-1 *did not show the degree of sequence divergence among the Brazilian states that was seen among different regions in the world. Mean π, π_*S*_, and π_*N *_within Brazilian states were significantly lower than the corresponding values within world regions (Figure 2). Likewise mean *d*, *d*_*S*_, and *d*_*N *_between Brazilian states were significantly lower than corresponding values between world regions (Figure 2). These results show that sequence divergence in *pvama-1 *among states in Brazil was low than that in comparisons of different Old World populations.

Similar results were obtained from estimation of pairwise *F*_*ST*_, which provides an index of the genetic differentiation between populations. *F*_*ST *_values among different world regions were often significantly greater than zero, indicating genetic differentiation between populations (Table 1). By contrast, estimates of *F*_*ST *_among Brazilian states were never significantly different from zero, indicating a lack of genetic differentiation among the Brazilian states (Table 2).

**Table 1:** *F*_*ST *_values at the *pvama-1 *locus among world regions.

	Brazil	Africa	India	Sri Lanka	China	S. Korea	Thailand	Morong	ADS	Palawan	PNG	Solomon Is.
**Africa**	0.233 **											
**India**	0.012	0.135										
**Sri Lanka**	0.101	0.230	-0.075									
**China**	0.124 **	0.194*	-0.040	-0.217								
**S. Korea**	0.078	0.641 **	0.120	0.393 **	0.278							
**Thailand**	0.198 ***	0.311 *	0.102 *	-0.016	0.197 *	0.401 **						
**Morong**	0.245 ***	0.131 ***	0.147 ***	0.124	0.194 ***	0.345 ***	0.187 **					
**ADS**	0.249 ***	0.118	0.135 **	0.065	0.115 *	0.450 ***	0.212 ***	0.144 ***				
**Palawan**	0.173 ***	0.098	0.065	0.009	0.069	0.332 ***	0.171 **	0.121 ***	-0.006			
**PNG**	0.200 ***	0.147 *	0.070 *	0.042	0.040	0.276 **	0.120 *	0.123 ***	0.176 ***	0.150 ***		
**Solomon Is.**	0.236 **	0.224 *	0.068 *	-0.083	-0.027	0.370 *	0.108	0.114	0.196 *	0.156 *	-0.089	
**Indonesia**	0.039	0.080	-0.065	-0.102	-0.076	0.170 *	0.165	0.106 *	0.102 *	0.030	0.030	-0.028

Significance of *F*_*ST*_: * P < 0.05; ** P < 0.01; *** P < 0.001.

**Table 2:** Estimates of *F*_*ST *_among Brazilian states^1^.

	Acre	Amazonas	Mato Grosso	Pará
**Amazonas**	-0.054			
**Mato Grosso**	-0.023	-0.026		
**Pará**	0.044	-0.040	0.014	
**Rondônia**	-0.042	-0.017	-0.007	0.045

^1^Roraima state was excluded because there was only one isolate from Roraima.

None of the *F*_*ST *_values was significant at the 5% level.

**Figure 3:**
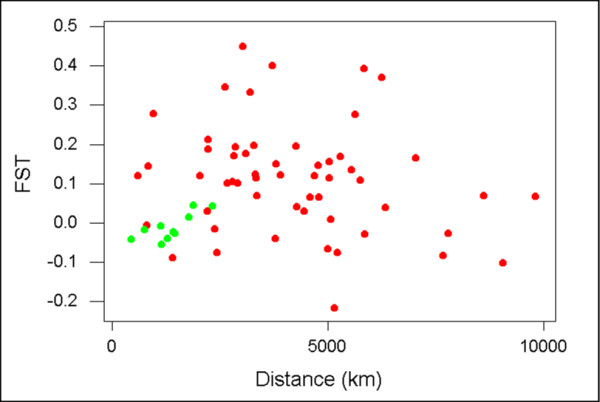
**Plot of *F*_*ST *_vs. geographical distance for Brazilian samples (*green*) and samples from Asia and Oceania (*red*). **In the data from Brazil, there was a significant correlation between *F*_*ST *_and geographical distance (r = 0.780; P < 0.01; randomization test). In the data from Asia and Oceania, there was not a significant correlation between *F*_*ST *_and geographical distance (r = -0.196; n.s.; randomization test).

We plotted *F*_*ST *_against the geographical distance between the sites where samples were collected separately for data from Brazil and data from Asia and Oceania (Figure 3). In the data from Asia and Oceania, there was not a significant correlation between *F*_*ST *_and geographical distance (r = -0.196; n.s.; randomization test; Figure 3). By contrast, in Brazil, there was a strong positive correlation between *F*_*ST *_and geographical distance (r = 0.780; P < 0.01; randomization test; Figure 3). The correlation coefficient for the Brazilian data was significantly different from that for the Asian and Oceanian data (p < 0.01; randomization test).

The range of geographical distances among the Brazilian samples (450–2340 km) overlapped only with the lower nine values from the Asian and Oceanian sample (range 600–2240; Figure 3). If we considered only the nine data points in the Asian and Oceanian sample that overlapped the Brazilian data, there was again no significant correlation between *F*_*ST *_and geographical distance (r = 0.091; n.s.; randomization test; Figure 3). Moreover, for the nine Asian and Oceanian comparisons with geographical distances comparable to those in Brazil, mean *F*_*ST *_(0.111) was significantly greater than that for the 10 Brazilian comparisons (mean *F*_*ST *_= -0.011; randomization test; P < 0.01).

## Discussion

Here, we characterized the polymorphic gene *pvama-1 *domain I in *Plasmodium vivax *isolates from patients in the Brazilian Amazon, where this species poses an important public health problem and compared those sequences with previously published sequences from the Old World. Although most branches in a phylogeny of *pvama-1 *sequences were not well resolved, it was clear that Brazilian sequences did not cluster separately from Old World sequences (Figure 1). This pattern supports the hypothesis that any reduction in population size accompanying the invasion of the Americas [12] was not so severe that only one or a few lineages of *pvama-1 *alleles survived in the New World. Rather, *pvama-1 *sequences from Brazil were found throughout the phylogenetic tree, consistent with the hypothesis that the alleles that became established in the New World represented a sample of worldwide genetic diversity at this locus. There was evidence of reduced genetic diversity at the *pvama-1 *locus in Brazil, consistent with some reduction in effective population size of *P. vivax *in the New World after its introduction.

There were very low values of *F*_*ST *_among the Brazilian states, with none being significantly greater than zero. The latter was in marked contrast to the Old World, particularly Southeast Asia, where high *F*_*ST *_values were consistently observed. Of course, the geographical distances among the Brazilian states sampled were low in comparison to many of the geographical distances among samples from Asia and Oceania (Figure 3). Nonetheless, mean *F*_*ST *_among the Brazilian samples was much lower than that among those samples from Asia and Oceania taken from comparable geographical distances. Thus, *pvama-1 *shows strikingly less geographical differentiation in Brazil than in Southeast Asia, consistent with a recent and rapid spread of the parasite in Brazil.

There is evidence that the polymorphism at the *ama-1 *locus is selectively maintained in *P. falciparum*, with host immune recognition likely being responsible for that selection [44]. Given the high polymorphism and prevalence of nonsynonymous polymorphisms at the *pvama-1 *locus, it seems likely that the same is true in *P. vivax *[20]. An alternative hypothesis to account for the reduced polymorphism in Brazilian *pvama-1 *sequences might be that selection at this locus has been relaxed in the New World. In the Brazilian Amazon, *P. vivax *has achieved high levels of infection in an ethnically diverse and rapidly growing host population [46]. If the selection on *pvama-1 *arises primarily from interaction with the human host immune system, it seems unlikely that selection would be relaxed under such circumstances. However, as long as the basis of natural selection on *pvama-1 *remains poorly known, it is impossible to rule out some role of natural selection in the pattern of sequence diversity observed in the New World.

In spite of the overall low *F*_*ST *_values in the Brazilian samples, there was evidence in Brazil of a strong positive relationship between *F*_*ST *_and geographical distance. By contrast, in the Old World, even though *F*_*ST *_values were high, there was no correlation between *F*_*ST *_and geographical distance. The latter was observed both in an extensive sample of populations from Asia and Oceania and when we examined only populations whose geographical distances were comparable to those among Brazilian states. The results from Brazil can be explained as reflecting effects of recent spread of the parasite, whereas those from the Old World appear to reflect a very ancient selectively maintained polymorphism. In the latter case, different populations are expected to show substantially different allelic frequency distributions due to divergent population histories, including the effects of genetic drift. Such a pattern is seen, for example, in the case of vertebrate major histocompatibility complex loci [50], at which high levels of polymorphism are maintained by balancing selection [51,52].

## Conclusion

Our results are consistent with the hypothesis that patterns of genetic diversity at highly polymorphic protein-coding loci of malaria parasites can show the effects of population history. Polymorphism at loci such as *pvama-1 *that are evidently subject to immune-driven selection may be an important factor in the epidemiology of infection by *P. vivax*. Understanding the factors governing the extent and pattern of polymorphism at such loci may thus have implications for the development of effective control strategies [53].

## Methods

### Study population

One hundred and five blood samples were collected from patients resident at Cuiabá district, capital of the state of Mato Grosso, northwestern Brazil (S 15°36'36", W 56°05'24"), where active malaria transmission does not occur. Patients had acquired malaria infection in different areas of the Brazilian Amazon comprising six different Brazilian states between April and August 1996 and between May 2001 and January 2006: 8 samples originated from Acre; 13 from Amazonas; 31 from Mato Grosso; 20 from Pará; 32 from Rondônia; and one from Roraima. Patient infections were confirmed by microscopic analysis of conventional thick smear method in a health facility (Hospital Júlio Müller, Universidade Federal de Mato Grosso). The age of patients ranged between 4 and 78 years old, with mean age of 37.6 ± 14.1.

### DNA extraction and amplification of *pvama-1*

Blood samples were stored in guanidine 4 M and kept at -20°C. The manufacturer's instructions for 300 μL whole blood extraction from Genomic DNA Purification Kit (Puregene^®^) were followed. The *pvama-1 *gene was amplified following a previously described protocol [20]. We added 0.26 pmoles of each primer [PvAR11 (5-TCC TAA ATT TTT ACG GGG GCA3) and PvAF11 (5-AGA ATT CCA GCT CCA AGA TG-3)], 0.2 mM of dNTPs, *Taq *buffer 1× (Phoneutria, MG, Brazil), 1.5 mM of MgCl_2_, 1.25 U of *Taq *polymerase (Phoneutria) and 5 μL of DNA totalizing 50 μL of mixture for the first round of amplification. One cycle of 95°C for 5 min, 2 cycles of 95°C for 30 s, 45°C for 50 s and 72°C for 40 s, 33 cycles of 95°C for 30 s, 55°C for 50 s and 72°C for 40 s, followed by 72°C for 10 min. The second round of amplification used the same conditions as the first one, except for the primers [PvAR11 and PvAF05 (5-GTA TCG TCA TAG AGA ATT CCG-3')] and quantity of DNA used (2 μL of the first round amplification product). The amplification conditions included one cycle of 95°C for 5 min, 2 cycles of 95°C for 30 s, 45°C for 50 s and 72°C for 40 s, 23 cycles of 95°C for 30 s, 55°C for 50 s and 72°C for 40 s, followed by 72°C for 10 min. The fragment size of approximately 400 bp was visualized on a 6% polyacrilamide gel.

### Purification of PCR products and *pvama-1 *sequencing

PCR products were purified using the GFX PCR DNA and Gel Band Purification Kit (Amersham Biosciences^®^, Little Chalfont, UK), following the manufacturer's instructions and were visualized on a 1% agarose gel to determine the DNA concentration. For the sequencing reaction, we used 4 μL of Dyenamic ET Dye Terminator Cycle Sequencing kit (Amersham Biosciences^®^) for MegaBace DNA Analysis Systems, 1 μL of primer and 5 μL of purified DNA. For each DNA sample, we created two sequences using both AR11 and AF05 primers.

### Sequence analysis

New sequences from Brazil were combined with a database of 215 sequences from Asia, Africa, and Oceania (see Additional file 1) and aligned with the ClustalW Software [54]. A 399-bp region was analyzed, corresponding to bases 322–720 (amino acid residues 108–240) of Genbank accession L27503. We used the MEGA 3.1 program [55] to estimate nucleotide diversity and evolutionary distances and to build phylogenetic trees by the neighbor-joining method [56], using the Jukes-Cantor distance [57]. The reliability of clustering patterns in the phylogenetic trees was assessed by bootstrapping [58]: 1000 bootstrap pseudo-samples were used. Before conducting the phylogenetic analysis, we tested for inter-allelic recombination using the maximum chi-square method [59] as implemented in the RDP2 program [60]. No recombination events were detected. The number of nucleotide substitutions per site (*d*) was estimated by Jukes and Cantor's method [57]. The numbers of synonymous nucleotide substitutions per synonymous site (*d*_*S*_) and the number of nonsynonymous substitutions per nonsynonymous site (*d*_*N*_) were estimated by Nei and Gojobori's method [61].

In order to examine patterns of nucleotide diversity within Brazil, we computed means of *d*, *d*_*S*_, and *d*_*N *_for all pairwise comparisons within and between the six Brazilian states from which we obtained sequences. Similarly, in order to examine patterns nucleotide diversity between regions throughout the world, we computed means of *d*, *d*_*S*_, and *d*_*N *_for all pairwise comparisons between the following geographic regions: Africa (5 sequences); Agusan del Sur, Philippines (abbreviation: ADS; 21 sequences), Brazil (93 sequences); China (8 sequences); India (15 sequences); Indonesia excluding Irian Jaya (5 sequences); Irian Jaya (1 sequence); Morong, Philippines (111 sequences); Myanmar (1 sequence); Palawan, Philippines (17 sequences); Papua New Guinea (abbreviation: PNG; 22 sequences); Solomon Islands (5 sequences); South Korea (4 sequences); Sri Lanka (3 sequences); Thailand (7 sequences); Vanuatu (2 sequences). Finally, in order to analyze nucleotide diversity within geographic regions other than Brazil, we computed means of *d*, *d*_*S*_, and *d*_*N *_for all pairwise comparisons within each of the above regions that was represented by at least two sequences. Following general usage, means of *d*, *d*_*S*_, and *d*_*N *_within populations were designated respectively π, π_*S*_, and π_*N *_.

Pairwise comparisons of *d*, *d*_*S*_, and *d*_*N *_are not statistically independent. Therefore, we tested hypotheses about the means of these variables using randomization (Monte Carlo) tests. Given *N *comparisons categorized (e.g., as within-region or between-region) by a classificatory variable *X*, in order to conduct simultaneous pairwise comparisons between categories with respect to the median of some continuous scalar variable *Y *measured on each of the *N *units (e.g., *d*, *d*_*S*_, or *d*_*N*_), we created 1000 pseudo data sets of *N *units each by randomly sampling (with replacement) independently from the vector of *X *values and from the vector of *Y *values. For a two-tailed test, the level of significance of the difference between two group medians was obtained by comparing the observed absolute difference with the distribution of absolute differences obtained for the corresponding groups in the 1000 pseudo data sets.

We used a similar randomization procedure to test the significance of correlation coefficients between pairwise measures of *F*_*ST *_and geographical distance. We created 1000 pseudo data sets by sampling at random from replacement in order to generate a null distribution against which observed values were compared. We used a similar procedure to test the equality of mean *F*_*ST *_in the Brazilian data with those from Asian and Oceanian populations of comparable geographic distance.

## Authors' contributions

PG carried out the molecular analyses, participated in statistical analyses, and drafted the manuscript. CJFF was responsible for acquisition of the data. ALH participated in design of the study and performed statistical analyses. EMB conceived the study and participated in its design and coordination.

## Supplementary Material

Additional file 1 List of the accession numbers of all sequences used in this study. The table lists the accession number of the sequences and the analyses in which each was used. Click here for file

Additional file 2 PvAMA-1 domain I polymorphic sites in Brazilian isolates compared with previously reported sequences. The table displays the polymorphisms (amino acid and nucleotide position) of *pvama-1 *domain I found in Brazilian isolates and compares them with previously reported sequences. Click here for file

Additional file 3 The nucleotide part represents the exclusive or particular features of Brazilian samples; the amino acid part represents the Brazilian haplotypes. This table illustrates the 27 haplotypes found in Brazilian samples, with amino acids residues and nucleotides positions. Click here for file
