# Supplementary material for: Polymorphism at the apical membrane antigen 1 locus reflects the world population history of Plasmodium vivax
Source: BMC Evol Biol. 2008 Apr 29;8:123. doi: 10.1186/1471-2148-8-123 (PMC2394524; doi:10.1186/1471-2148-8-123)
Supplement: Additional file 1 — List of the accession numbers of all sequences used in this study. The table lists the accession number of the sequences and the analyses in which each was used. [file 1471-2148-8-123-S1.doc]

Supplementary Table 1 – List of the accession numbers of all sequences used in this study.

| Sample | Accession number | Used in | Sample | Accession number | Used in |
| --- | --- | --- | --- | --- | --- |
| BR001 | EF031154 | 1,2 | BR054 | EF031207 | 1,2 |
| BR002 | EF031155 | 1,2 | BR055 | EF031208 | 1,2 |
| BR003 | EF031156 | 1,2 | BR056 | EF031209 | 1,2 |
| BR004 | EF031157 | 1,2 | BR057 | EF031210 | 1,2 |
| BR005 | EF031158 | 1,2 | BR058 | EF031211 | 1,2 |
| BR006 | EF031159 | 1 | BR059 | EF031212 | 1,2 |
| BR007 | EF031160 | 1,2 | BR060 | EF031213 | 1,2 |
| BR008 | EF031161 | 1,2 | BR061 | EF031214 | 1,2 |
| BR009 | EF031162 | 1,2 | BR062 | EF031215 | 1 |
| BR010 | EF031163 | 1,2 | BR063 | EF031216 | 1,2 |
| BR011 | EF031164 | 1,2 | BR064 | EF057446 | 1,2 |
| BR012 | EF031165 | 1,2 | BR065 | EF057447 | 1,2 |
| BR013 | EF031166 | 1,2 | BR066 | EF057448 | 1,2 |
| BR014 | EF031167 | 1,2 | BR067 | EF057449 | 1,2 |
| BR015 | EF031168 | 1,2 | BR068 | EF057450 | 1 |
| BR016 | EF031169 | 1,2 | BR069 | EF057451 | 1,2 |
| BR017 | EF031170 | 1,2 | BR070 | EF057452 | 1,2 |
| BR018 | EF031171 | 1,2 | BR071 | EF057453 | 1,2 |
| BR019 | EF031172 | 1,2 | BR072 | EF057454 | 1,2 |
| BR020 | EF031173 | 1,2 | BR073 | EF057455 | 1,2 |
| BR021 | EF031174 | 1,2 | BR074 | EF057456 | 1,2 |
| BR022 | EF031175 | 1,2 | BR075 | EF057457 | 1,2 |
| BR023 | EF031176 | 1,2 | BR076 | EF057458 | 1,2 |
| BR024 | EF031177 | 1,2 | BR077 | EF057459 | 1,2 |
| BR025 | EF031178 | 1,2 | BR078 | EF057460 | 1,2 |
| BR026 | EF031179 | 1,2 | BR079 | EF057461 | 1,2 |
| BR027 | EF031180 | 1,2 | BR080 | EF057462 | 1,2 |
| BR028 | EF031181 | 1 | BR081 | EF057463 | 1,2 |
| BR029 | EF031182 | 1,2 | BR082 | EF057464 | 1,2 |
| BR030 | EF031183 | 1,2 | BR083 | EF057465 | 1,2 |
| BR031 | EF031184 | 1 | BR084 | EF057466 | 1,2 |
| BR032 | EF031185 | 1,2 | BR085 | EF057467 | 1,2 |
| BR033 | EF031186 | 1,2 | BR086 | EF057468 | 1,2 |
| BR034 | EF031187 | 1,2 | BR087 | EF057469 | 1,2 |
| BR035 | EF031188 | 1,2 | BR088 | EF057470 | 1,2 |
| BR036 | EF031189 | 1,2 | BR089 | EF057471 | 1,2 |
| BR037 | EF031190 | 1,2 | BR090 | EF057472 | 1,2 |
| BR038 | EF031191 | 1 | BR091 | EF057473 | 1,2 |
| BR039 | EF031192 | 1,2 | BR092 | EF057474 | 1,2 |
| BR040 | EF031193 | 1,2 | BR093 | EF057475 | 1,2 |
| BR041 | EF031194 | 1,2 | BR094 | EF057476 | 1,2 |
| BR042 | EF031195 | 1,2 | BR095 | EF057477 | 1,2 |
| BR043 | EF031196 | 1,2 | BR096 | EF057478 | 1,2 |
| BR044 | EF031197 | 1,2 | BR097 | EF057479 | 1,2 |
| BR045 | EF031198 | 1,2 | BR098 | EF057480 | 1,2 |
| BR046 | EF031199 | 1,2 | BR099 | EF057481 | 1,2 |
| BR047 | EF031200 | 1,2 | BR100 | EF057482 | 1 |
| BR048 | EF031201 | 1,2 | BR101 | EF057483 | 1 |
| BR049 | EF031202 | 1,2 | BR102 | EF057484 | 1 |
| BR050 | EF031203 | 1,2 | BR103 | EF057485 | 1 |
| BR051 | EF031204 | 1,2 | BR104 | EF057486 | 1 |
| BR052 | EF031205 | 1,2 | BR105 | EF057488 | 1 |
| BR053 | EF031206 | 1,2 |  |  |  |

1 = Sequences combined with a database of sequences from throughout the world and aligned with the ClustalW Software for comparisons. 2 = Sequences used to calculate nucleotide diversity and evolutionary distances and to build phylogenetic trees by the Neighbor Joining method [43], to calculate the number of nucleotide substitutions per site (d) using Jukes and Cantor’s method [45], the numbers of synonymous nucleotide substitutions per synonymous site (*d_S_*) and the number of nonsynonymous substitutions per nonsynonymous site (*d_N_*), estimated by Nei and Gojobori’s method [46], and to calculate Linkage Disequilibrium (LD) with Arlequim 3.1 software.
