# Supplementary material for: Polymorphism at the apical membrane antigen 1 locus reflects the world population history of Plasmodium vivax
Source: BMC Evol Biol. 2008 Apr 29;8:123. doi: 10.1186/1471-2148-8-123 (PMC2394524; doi:10.1186/1471-2148-8-123)
Supplement: Additional file 2 — PvAMA-1 domain I polymorphic sites in Brazilian isolates compared with previously reported sequences. The table displays the polymorphisms (amino acid and nucleotide position) of pvama-1 domain I found in Brazilian isolates and compares them with previously reported sequences. [file 1471-2148-8-123-S2.doc]

|  | 92 (274,275) | 100 (298) | 107 (320,321*) | 111 (333) | 112 (335,336) | 120 (359,360) | 130 (390*) | 132 (394) | 133 (397) | 140 (418) | 141 (422) | 145 (434) | 155 (463) | 172 (514) | 188 (564) | 189 (565, 567) | 190 (568) | 191 (572) | 193 (577) | 210 (628) | 218 (652) | 227 (680, 682) | 228 (683, 684) | 237 (709) | 241 (721) | 248 (744*) |
| --- | --- | --- | --- | --- | --- | --- | --- | --- | --- | --- | --- | --- | --- | --- | --- | --- | --- | --- | --- | --- | --- | --- | --- | --- | --- | --- |
| Brazil | G/P | C/G | A/D | L/F | K/R/T | K/R | K*/N | D/N | D | I/L | A/E | A/E | K/E | A | K | E/K/N | K/Q | T | H/Y | P/S | V | E/V | D/S | K/Q | N/Y | P/P* |
| Africa^12^ | - | - | - | L | K/R/T | K/R | N | D/N | D | I/L | A | A/E | K | A | K | E | K | T | H | S | V | E | S | K | N | - |
| India^12,31^ | - | - | A/D/N | L | K/R/T | K/R | K*/N | D/N | D | I/L | A/E | A/E | K | A | K | E/K/N | E/K/Q | T | H/Y | P/S | V | E/V | D/S | K | N | - |
| Sri Lanka^12,30^ | - | - | A/D/N | L | K/T | K/R | N | D/N | D | I/L | A/E | A/E | K | A | N | E/K | E/K | T | H/Y | P/S | V/L | E/V | D/ | K | N | - |
| Thailand^12^ | - | - | - | L | K/T | K/R | N | D/N | D | I | A | A | K | A | K | E/K | E/K | T | H | P/S | V | E/V | D/S | K | N | - |
| China^12^ | - | - | - | L | K/R/T | R | N | D/N | D | I/L | A/E | A/E | K | A | K | E/K | K | T/K | H | P/S | V | E/V | D/S | x | x | - |
| South Korea^27,28^ | - | G | A | L | T | R | N | D | D | L | E | A | K | A | K | E/K | K | T | H | P | V | E | S | K | N | P |
| Indonesia^12^ | - | - | - | L | K/T | R/S | K/N | D/N | D/N | I/L | A/E | A/E | K | A | K | E/N | K | T | H | P/S | V | E/V | D/S | K | N | - |
| ADS^12^ | - | - | - | L | K/T | R | K/N | D/N | D | I/L | A/E | A/E | K | A | K | E/K/N | E/K | T | H | P/S | L/V | E/V | D/S | K | N | - |
| Morong^12^ | - | - | - | L | K/T | R | K/N | D/N | D | I/L | A/E | A/E | K | A | K | E/K/N | E/K | T | H | P/S | L/V | E/V | D/S | K | N | - |
| Palawan^12^ | - | - | - | L | K/T | R | K/N | D/N | D | I/L | A/E | A/E | K | A | K | E/K | E/K | T | H/Y | P/S | L/V | E/V | D/S | K | N | - |
| P.N.G.^12^ | - | - | - | L | K/R/T | R/S | K/N | D/N | D/N | I/L | A/E | A/E | K | A/T | K | E/K/N | E/K | T | H | S | V | E/V | D/S | K | N | - |
| Sol. Isl.^12^ | - | - | - | L | K/T | R/S | K/N | D/N | D/N | I/L | A/E | A/E | K | A/T | K | E/K | E/K | T | H | S | V | E/V | D/S | K | N | - |

Additional file 2. PvAMA-1 domain I polymorphic sites in Brazilian isolates compared with previously reported sequences.

Grey highlights = not found in Brazilian sequences. Underline = found only in Brazilian sequences. Numbers indicate amino acid sites; numbers is parentheses indicate nucleotide sites. “-“ = not available information.

Grey highlights = not found in Brazilian sequences; Underline = only found in Brazilian sequences
