# Supplementary material for: Polymorphism at the apical membrane antigen 1 locus reflects the world population history of Plasmodium vivax
Source: BMC Evol Biol. 2008 Apr 29;8:123. doi: 10.1186/1471-2148-8-123 (PMC2394524; doi:10.1186/1471-2148-8-123)
Supplement: Additional file 3 — The nucleotide part represents the exclusive or particular features of Brazilian samples; the amino acid part represents the Brazilian haplotypes. This table illustrates the 27 haplotypes found in Brazilian samples, with amino acids residues and nucleotides positions. [file 1471-2148-8-123-S3.doc]

Additional file 3: The nucleotide part represents the exclusive or particular features of Brazilian samples; the amino acid part represents the Brazilian haplotypes

|  | 320 | 321 | 333 | 335 | 336 | 359 | 390 | 394 | 418 | 422 | 424 | 434 | 463 | 565 | 567 | 568 | 577 | 628 | 680 | 682 | 683 | 684 | 709 |  |  | 107 | 111 | 112 | 120 | 130 | 132 | 140 | 141 | 142 | 145 | 155 | 189 | 190 | 193 | 210 | 227 | 228 | 237 | Brazilian states |
| --- | --- | --- | --- | --- | --- | --- | --- | --- | --- | --- | --- | --- | --- | --- | --- | --- | --- | --- | --- | --- | --- | --- | --- | --- | --- | --- | --- | --- | --- | --- | --- | --- | --- | --- | --- | --- | --- | --- | --- | --- | --- | --- | --- | --- |
| Hap1 (16) | C | T | A | C | C | G | T | A | T | A | A | C | A | A | A | C | C | C | A | A | G | C | A |  |  | A | L | T | R | N | N | L | E | N | A | K | K | Q | H | P | E | S | K | AM/MT/PA/RO/RR |
| Hap2 (13) | . | . | . | . | . | . | A | G | . | C | . | A | . | G | . | A | . | . | . | . | . | . | . |  |  | . | . | . | . | K | D | . | A | . | E | . | E | K | . | . | . | . | . | AC/AM/PA/RO |
| Hap3 (6) | . | . | . | . | . | . | . | . | A | C | . | . | . | G | . | A | . | . | . | . | . | . | . |  |  | . | . | . | . | . | . | I | A | . | . | . | E | K | . | . | . | . | . | AM/MT/PA |
| Hap4 (4) | . | . | . | . | . | . | . | . | . | . | . | . | . | G | . | A | . | . | . | . | . | . | . |  |  | . | . | . | . | . | . | . | . | . | . | . | E | K | . | . | . | . | . | AC/RO |
| Hap5 (3) | . | . | . | . | . | . | A | G | . | C | . | . | . | G | . | A | T | T | . | . | . | . | . |  |  | . | . | . | . | K | D | . | A | . | . | . | E | K | Y | S | . | . | . | MT/RO |
| Hap6 (3) | . | . | . | . | . | . | A | G | . | . | . | . | . | G | . | A | T | T | . | . | . | . | . |  |  | . | . | . | . | K | D | . | . | . | . | . | E | K | Y | S | . | . | . | MT/PA/RO |
| Hap7(2) | . | . | . | . | . | . | A | G | . | C | . | A | . | G | . | A | . | . | T | G | A | T | . |  |  | . | . | . | . | K | D | . | A | . | E | . | E | K | . | . | V | D | . | MT |
| Hap8 (2) | . | . | . | . | . | . | . | . | A | C | . | . | . | G | . | A | . | . | . | . | . | . | C |  |  | . | . | . | . | . | . | I | A | . | . | . | E | K | . | . | . | . | Q | MT |
| Hap9 (2) | . | . | . | . | . | . | . | . | A | C | . | A | . | G | . | A | . | . | . | . | . | . | . |  |  | . | . | . | . | . | . | I | A | . | E | . | E | K | . | . | . | . | . | PA |
| Hap10 (1) | . | . | . | . | . | . | . | . | A | C | G | A | G | G | . | A | . | . | . | . | . | . | C |  |  | . | . | . | . | . | . | I | A | D | E | E | E | K | . | . | . | . | Q | MT |
| Hap11 (1) | . | . | . | . | . | . | . | . | A | . | . | . | . | G | . |  | . | . | . | . | . | . | . |  |  | . | . | . | . | . | . | I | . | . | . | . | E | . | . | . | . | . | . | MT |
| Hap12 (1) | . | . | . | . | . | . | . | . | . | . | . | . | . | . | . |  | . | . | . | . | . | . | C |  |  | . | . | . | . | . | . | . | . | . | . | . | . | . | . | . | . | . | Q | MT |
| Hap13 (1) | . | . | . | . | . | . | A | G | . | . | . | . | . | G | . | A | T | T | . | . | . | . | C |  |  | . | . | . | . | K | D | . | . | . | . | . | E | K | Y | S | . | . | Q | RO |
| Hap14 (1) | . | . | . | . | . | . | A | G | . | C | . | . | . | G | . | A | T | T | . | . | . | . | C |  |  | . | . | . | . | K | D | . | A | . | . | . | E | K | Y | S | . | . | Q | MT |
| Hap15 (1) | . | . | . | . | . | . | . | G | . | C | . | . | . | G | . | A | T | T | . | . | . | . | . |  |  | . | . | . | . | . | D | . | A | . | . | . | E | K | Y | S | . | . | . | PA |
| Hap16 (1) | . | . | . | . | . | . | . | . | A | C | . | . | . | G | . |  | . | . | . | . | . | . | . |  |  | . | . | . | . | . | . | I | A | . | . | . | E | . | . | . | . | . | . | PA |
| Hap17 (1) | . | . | . | . | . | . | . | . | . | . | . | . | . | G | . | A | T | T | . | . | . | . | . |  |  | . | . | . | . | . | . | . | . | . | . | . | E | K | Y | S | . | . | . | RO |
| Hap18 (1) | . | . | . | . | . | . | . | . | . | . | . | . | . | G | . | A | . | . | . | . | . | . | C |  |  | . | . | . | . | . | . | . | . | . | . | . | E | K | . | . | . | . | Q | RO |
| Hap19 (1) | . | . | T | . | . | . | . | . | . | . | . | . | . | . | . |  | . | . | . | . | . | . | . |  |  | . | F | . | . | . | . | . | . | . | . | . | . | . | . | . | . | . | . | MT |
| Hap20 (14) | A | . | . | . | . | . | A | G | . | . | . | . | . | G | T | A | . | . | T | G | A | T | . |  |  | D_1_ | . | . | . | K | D | . | . | . | . | . | N | K | . | . | V | D | . | AC/AM/MT/PA/RO |
| Hap21 (1) | A | . | . | . | . | . | A | G | . | . | . | . | . | G | T | A | . | . | T | G | A | T | C |  |  | D_1_ | . | . | . | K | D | . | . | . | . | . | N | K | . | . | V | D | Q | RO |
| Hap22 (1) | A | . | . | . | . | . | A | G | . | . | . | . | . | G | T | A | . | . | . | . | . | . | . |  |  | D_1_ | . | . | . | K | D | . | . | . | . | . | N | K | . | . | . | . | . | RO |
| Hap23 (5) | A | C | . | A | A | . | . | . | A | C | . | . | . | G | . | A | . | . | . | . | . | . | . |  |  | D_2_ | . | K | . | . | . | I | A | . | . | . | E | K | . | . | . | . | . | MT/PA/RO |
| Hap24 (2) | A | C | T | A | A | . | . | . | A | C | . | . | . | G | . | A | . | . | . | . | . | . | . |  |  | D_2_ | F | K | . | . | . | I | A | . | . | . | E | K | . | . | . | . | . | AM/PA |
| Hap25 (5) | A | C | . | G | A | A | . | . | A | C | . | . | . | G | . | A | . | . | . | . | . | . | . |  |  | D_2_ | . | R | K | . | . | I | A | . | . | . | E | K | . | . | . | . | . | AC/AM/PA |
| Hap26 (3) | A | C | . | G | A | A | . | . | A | C | . | . | . | G | . | A | . | . | T | G | A | T | . |  |  | D_2_ | . | R | K | . | . | I | A | . | . | . | E | K | . | . | V | D | . | AM/PA/RO |
| Hap27 (1) | A | C | . | G | A | A | . | . | A | C | . | . | . | G | . | A | . | . | . | . | . | . | C |  |  | D_2_ | . | R | K | . | . | I | A | . | . | . | E | K | . | . | . | . | Q | RO |

D_1_ = GAT; D_2_ = GAC

Table 2: Comparison between Brazilian polymorphism with other places

|  | 92 (274,275) | 100 (298) | 107 (320,321*) | 111 (333) | 112 (335,336) | 120 (359,360) | 130 (390*) | 132 (394) | 133 (397) | 140 (418) | 141 (422) | 142 (424) | 145 (434) | 155 (463) | 172 (514) | 189 (565, 567) | 190 (568) | 191 (572) | 193 (577) | 210 (628) | 218 (652) | 227 (680,682) | 228 (683,684) | 237 (709) | 241 (721) | 248 (744*) |  |
| --- | --- | --- | --- | --- | --- | --- | --- | --- | --- | --- | --- | --- | --- | --- | --- | --- | --- | --- | --- | --- | --- | --- | --- | --- | --- | --- | --- |
| Brazil | G/P | C/G | A/D | L/F | K/R/T | K/R | K*/N | D/N | D | I/L | A/E | N/D | A/E | K/E | A | E/K/N | K/Q | T | H/Y | P/S | V | E/V | D/S | K/Q | N/Y | P/P* | Figtree et al., 2000 |
| Africa | . | . | . | L | K/R/T | K/R | N | D/N | D | I/L | A | N | A/E | K | A | E | K | T | H | S | V | E | S | K | N | . | Figtree et al., 2000 |
| Índia | . | . | . | L | K/R/T | K/R | K*/N | D/N | D | I/L | A/E | N | A/E | K | A | E/K/N | E/Q | T | H | P/S | V | E/V | D/S | K | N | . | Figtree et al., 2000 |
| Sri Lanka | . | . | . | L | K/T | K/R | N | D/N | D | I/L | A/E | N | A/E | K | A | E/K | E/K | T | H | P/S | V | E/V | D/ | K | N | . | Figtree et al., 2000 |
| Thailand | . | . | . | L | K/T | K/R | N | D/N | D | I | A | N | A | K | A | E/K | E/K | T | H | P/S | V | E/V | D/S | K | N | . | Figtree et al., 2000 |
| China | . | . | . | L | K/R/T | R | N | D/N | D | I/L | A/E | N | A/E | K | A | E/K | K | T/K | H | P/S | V | E/V | D/S | . | . | . | Han et al., 2002; Chung et al., 2002 |
| South Koreia | . | G | A | L | T | R | N | D | D | L | E | N | A | K | A | E/K | K | T | H | P | V | E | S | K | N | P | Figtree et al., 2000 |
| Indonesia | . | . | . | L | K/T | R/S | K/N | D/N | D/N | I/L | A/E | N | A/E | K | A | E/N | K | T | H | P/S | V | E/V | D/S | K | N | . | Figtree et al., 2000 |
| ADS | . | . | . | L | K/T | R | K/N | D/N | D | I/L | A/E | N | A/E | K | A | E/K/N | E/K | T | H | P/S | L/V | E/V | D/S | K | N | . | Figtree et al., 2000 |
| Morong | . | . | . | L | K/T | R | K/N | D/N | D | I/L | A/E | N | A/E | K | A | E/K/N | E/K | T | H | P/S | L/V | E/V | D/S | K | N | . | Figtree et al., 2000 |
| Palawan | . | . | . | L | K/T | R/S | K/N | D/N | D | I/L | A/E | N | A/E | K | A | E/K | E/K | T | H/Y | P/S | L/V | E/V | D/S | K | N | . | Figtree et al., 2000 |
| P.N. Guinea | . | . | . | L | K/R/T | R | K/N | D/N | D/N | I/L | A/E | N | A/E | K | A/T | E/K/N | E/K | T | H | S | V | E/V | D/S | K | N | . | Figtree et al., 2000 |
| Sol. Islands | . | . | . | L | K/T | R/S | K/N | D/N | D/N | I/L | A/E | N | A/E | K | A/T | E/K | E/K | T | H | S | V | E/V | D/S | K | N | . | Figtree et al., 2000 |
